# Metagenomic Exploration of the Marine Sponge *Mycale hentscheli* Uncovers Multiple Polyketide-Producing Bacterial Symbionts

**DOI:** 10.1128/mBio.02997-19

**Published:** 2020-03-24

**Authors:** Mathew A. Storey, Sarah K. Andreassend, Joe Bracegirdle, Alistair Brown, Robert A. Keyzers, David F. Ackerley, Peter T. Northcote, Jeremy G. Owen

**Affiliations:** a School of Biological Sciences, Victoria University of Wellington, Wellington, New Zealand; b School of Chemical and Physical Sciences, Victoria University of Wellington, Wellington, New Zealand; c Centre for Biodiscovery, School of Biological Sciences, Victoria University of Wellington, Wellington, New Zealand; d Maurice Wilkins Centre for Molecular Biodiscovery, Auckland, New Zealand; e The Ferrier Institute, Victoria University of Wellington, Wellington, New Zealand; McMaster University

**Keywords:** biosynthesis, metagenomics, polyketides, secondary metabolism, symbiosis

## Abstract

Marine sponges have been a prolific source of unique bioactive compounds that are presumed to act as a deterrent to predation. Many of these compounds have potential therapeutic applications; however, the lack of efficient and sustainable synthetic routes frequently limits clinical development. Here, we describe a metagenomic investigation of Mycale hentscheli, a chemically gifted marine sponge that possesses multiple distinct chemotypes. We applied shotgun metagenomic sequencing, hybrid assembly of short- and long-read data, and metagenomic binning to obtain a comprehensive picture of the microbiome of five specimens, spanning three chemotypes. Our data revealed multiple producing species, each having relatively modest secondary metabolomes, that contribute collectively to the chemical arsenal of the holobiont. We assembled complete genomes for multiple new genera, including two species that produce the cytotoxic polyketides pateamine and mycalamide, as well as a third high-abundance symbiont harboring a proteusin-type biosynthetic pathway that appears to encode a new polytheonamide-like compound. We also identified an additional 188 biosynthetic gene clusters, including a pathway for biosynthesis of peloruside. These results suggest that multiple species cooperatively contribute to defensive symbiosis in *M. hentscheli* and reveal that the taxonomic diversity of secondary-metabolite-producing sponge symbionts is larger and richer than previously recognized.

## Abstract (precis)

*Mycale hentscheli* is a marine sponge that is rich in bioactive small molecules. Here, we use direct metagenomic sequencing to elucidate highly complete and contiguous genomes for the major symbiotic bacteria of this sponge. We identify complete biosynthetic pathways for the three potent cytotoxic polyketides which have previously been isolated from *M. hentscheli*. Remarkably, and in contrast to previous studies of marine sponges, we attribute each of these metabolites to a different producing microbe. We also find that the microbiome of *M. hentscheli* is stably maintained among individuals, even over long periods of time. Collectively, our data suggest a cooperative mode of defensive symbiosis in which multiple symbiotic bacterial species cooperatively contribute to the defensive chemical arsenal of the holobiont.

## INTRODUCTION

The production of specialized cytotoxic metabolites by the symbiotic bacteria of sessile marine invertebrates is believed to be a key driver for maintaining symbiosis (1). In this relationship, the microbe provides a defensive advantage to the host by deterring predation and preventing fouling and in return is provided with a hospitable environment (2–5). The ecological benefits of this interaction, when maintained over an evolutionary time frame, appear to have driven interdependence and exclusivity between some host-symbiont pairs. In several cases, this has resulted in symbiont genome specialization, reduction, and degradation, while secondary metabolite biosynthesis remains functional and under positive selective pressure (1, 6, 7). In addition to their important ecological roles, the exquisite potency and specificity against eukaryotic cellular pathways that are often exhibited by marine invertebrate secondary metabolites have fueled significant efforts toward discovery and development of these compounds as chemotherapeutic agents (8–10). However, challenges associated with the sustainable supply of lead compounds have frequently thwarted clinical development (10–12).

Marine sponges (phylum Porifera) have proven a particularly rich source for the discovery of structurally unique secondary metabolites that possess potent biological activities (8, 13, 14). Metagenomic analysis of sponge microbiomes has been used to verify the bacterial origin of several polyketide and modified peptide secondary metabolites (15–22). Of particular significance in this context is the recent discovery of the phylum Tectomicrobia, a widely distributed and biosynthetically gifted taxon, members of which are the producers of almost all of the compounds previously described from sponges of the genus *Theonella* (23). However, the total number of sponge microbiomes examined is still relatively low, and that of studies linking genes to chemistry is even lower. It is therefore likely that additional symbiotic bacterial genera and the symbiotic systems that produce complex natural products remain to be discovered (20).

Many of the most potent and structurally distinct secondary metabolites that have been isolated from marine invertebrates are polyketide derived, and metagenomic studies have revealed that these are often the products of *trans*-acting acyltransferase (*trans*-AT) polyketide synthase (PKS) systems (24). In *trans*-AT PKS, modules lack the integrated AT domains that are found in *cis*-AT PKS and instead utilize at least one freestanding AT enzyme that catalyzes acyl transfer for multiple modules. *trans*-AT PKS gene clusters frequently have unusual cluster architectures, and this is particularly true of the pathways that have been elucidated from marine symbionts (1, 6, 25), where copy number variation, cluster fragmentation, noncanonical modules, deviation from colinearity, and the presence of repeated or nonfunctional domains within modules are all common features. This flexibility and diversity of cluster configuration are reflected by the structural diversity of the densely functionalized chemical products produced by these biosynthetic gene clusters (BGCs) (24, 26).

Sponges belonging to the genus *Mycale* have yielded numerous bioactive metabolites (27–35), but as yet there are no studies that conclusively link this chemistry to a producing bacterium. Mycale hentscheli is an especially metabolically diverse organism and is the source of three potent cytotoxic polyketides and their congeners: pateamine (compound 1) (36), peloruside (compound 2) (33), and mycalamide (compound 3) (34). Each of these compounds possesses potent cytotoxic activity, exerted via a different cellular target. Compounds 1 to 3 have been isolated in various proportions from different *M. hentscheli* samples, and their structures suggest that they are likely to be the products of bacterial *trans-*AT PKS/nonribosomal peptide synthetase (NRPS)-type biosynthetic systems.

As a first step toward developing a sustainable biosynthetic route toward production of these molecules, we employed metagenomic sequencing to examine the microbiome of *M. hentscheli*, seeking the biosynthetic pathways and producing organism(s) for each of the major metabolites. In a hybrid assembly of PacBio and Illumina data, we identified complete and contiguous BGCs, in their full genomic contexts, for both mycalamide and pateamine. Additional BGCs were also discovered that appear to encode molecules not previously isolated from *M. hentscheli*, including a ribosomally synthesized and posttranslationally modified peptide (RiPP) gene cluster that likely encodes a new polytheonamide-like metabolite. We also discovered the BGC for peloruside, although we were not able to assign this to a producing organism. In total, we sequenced five *M. hentscheli* specimens, and we were able to assemble 26 complete high-quality metagenome-assembled genomes (MAGs), of >85% completeness (average 94.4%) and <15% contamination (average 2.8%), across the pan-metagenome. Both the mycalamide and pateamine producers were found to be taxonomically distinct from any cultivated or known uncultivated species, including the best-studied secondary-metabolite-producing sponge symbionts *Entotheonella* species, and were each sufficiently distal to represent novel genera. In the case of the pateamine producer, the contiguous genome afforded by the hybrid metagenome assembly (98.7% of the MAG being captured in a single contig) was essential for resolving the true architecture of the pateamine gene cluster. Collectively, our data suggest a cooperative mode of symbiosis that starkly contrasts with the single-producer paradigm observed for the Theonellidae.

## RESULTS AND DISCUSSION

### Metagenomic analysis of the *Mycale hentscheli* microbiome.

**FIG 1:**
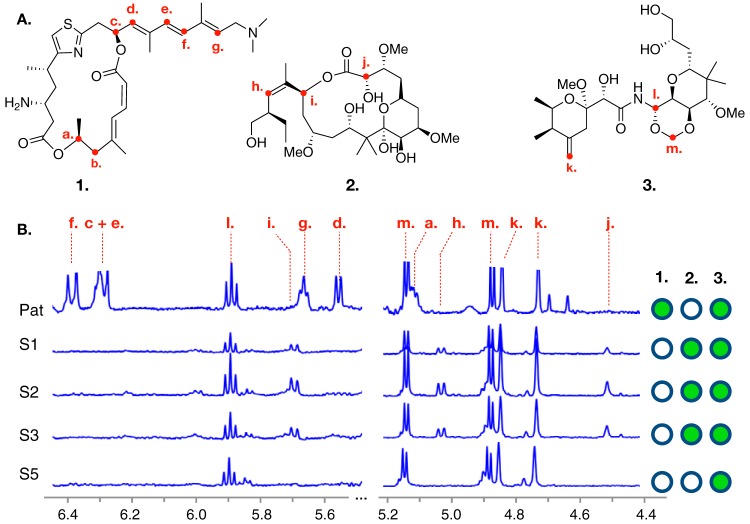
Structures of major metabolites and chemotyping of *Mycale hentscheli* specimens. (A) Structures for each of the cytotoxic polyketides previously isolated from *M. hentscheli* are shown. These are pateamine A (compound 1), peloruside A (compound 2), and mycalamide A (compound 3). Red labels indicate positions of protons whose shifts were diagnostic of compound presence during chemotyping experiments. (B) Selected regions of ^1^H NMR spectra for each of the five specimens examined in this study. Diagnostic peaks for the presence of each compound are labeled with dashed red lines, and the letters above these match the positions of protons in panel A. The right-hand panel indicates the compounds that were determined to be present in each specimen.

Our search for the pateamine, mycalamide, and peloruside BGCs began with a specimen of *M. hentscheli* collected in November 2014 from Capsize Point in the South Island of New Zealand (specimen MH-PAT). Detection and quantitation of secondary metabolites in this specimen were carried out using ^1^H nuclear magnetic resonance (NMR) spectroscopy of reversed-phase fractionated methanolic extracts and revealed signals characteristic of both compounds 1 and 3 (Fig. 1). We were not able to detect compound 2 in this sample using ^1^H NMR spectroscopy; however, subsequent examination using liquid chromatography-tandem mass spectrometry (LC-MS/MS) revealed that this metabolite was present (see Fig. S1 in the supplemental material). Differential centrifugation and microscopic examination of our sample using previously described differential centrifugation protocols failed to detect any evidence of bacteria with fluorescent or filamentous morphologies, leading us to hypothesize that the producer(s) in *M. hentscheli* might not belong to the phylum Tectomicrobia. This hypothesis was supported by phylogenetic analysis of metagenome sequence data in our subsequent experiments; however, we cannot rule out the possibility that Tectomicrobia were present but were lost during our sample preparation.

10.1128/mBio.02997-19.1 FIG S1 (A) LC-MS-extracted chromatogram of peloruside standard. (B) LC-MS-extracted chromatogram of crude methanolic extract from sample PAT showing compound with congruent mass and retention time. Download FIG S1, PDF file, 0.1 MB. Copyright © 2020 Storey et al. 2020 Storey et al. This content is distributed under the terms of the Creative Commons Attribution 4.0 International license.

**FIG 2:**
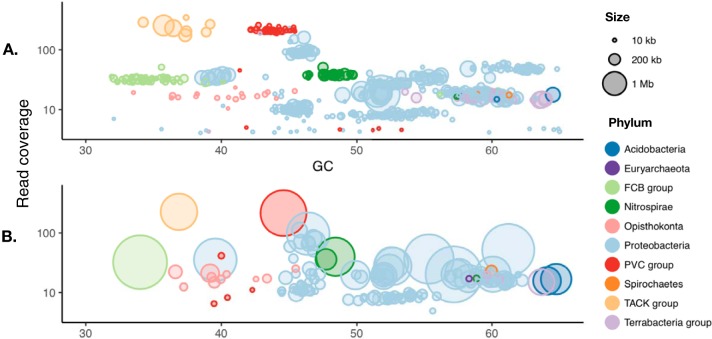
PacBio data facilitate contiguous assembly of the major microbiome members in an *M. hentscheli* specimen. (A) Contig size and predicted superphylum-level taxonomy for an assembly conducted using Illumina data only. (B) A hybrid assembly incorporating PacBio and Illumina data possessing greatly improved contiguity of assembled genomes for the major microbial members present in the consortium. GC, percent GC content.

As neither the phylogenetic identity nor localization of the producing microbes within the sponge was known, we sought to sample the microbiome as completely as possible by extracting metagenomic DNA from a longitudinal section through an entire individual leuconoid. Initially, we obtained 10 Gbp of PE250 Illumina sequence data to permit an explorative low-depth pilot assembly (assembly MH-Pat-low). These reads were assembled using IDBA-UD (37), yielding a total of 328.9 Mb of assembled genome sequence distributed over 68,286 contigs (≥1,000 bp), with an *N*_50_ of 6,716 bp (Data Set S1). All contigs larger than 2 kb from the assembly were analyzed using antiSMASH4 (38) to identify and annotate BGCs, and where possible, superphylum-level taxonomy was assigned to contigs based on homology of conserved essential genes (39) (Fig. 2).

10.1128/mBio.02997-19.7 DATA SET S1 Summary statistics for each of the metagenome assemblies described in this study. Download Data Set S1, XLSX file, 0.01 MB. Copyright © 2020 Storey et al. 2020 Storey et al. This content is distributed under the terms of the Creative Commons Attribution 4.0 International license.

**FIG 3:**
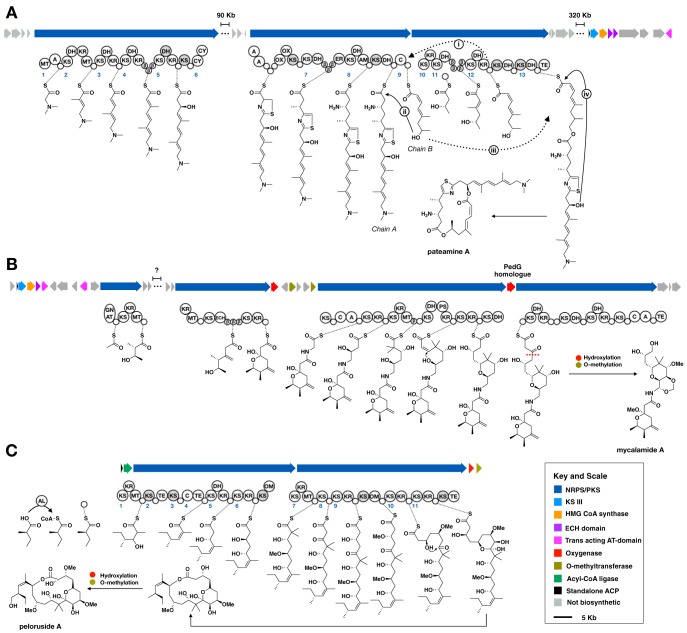
Biosynthetic models for the cytotoxic polyketides of *M. hentscheli*. (A) Biosynthetic model for pateamine. Dashed arrows indicate chain transfer events; solid arrows indicate esterification events. These are numbered according to the description in the main text. (B) Biosynthetic model for mycalamide; the red dashed line indicates the putative site of oxidative chain termination catalyzed by the pathway-encoded PedG homolog. (C) Biosynthetic model for peloruside. For all panels, biosynthetic predictions were deduced from domain arrangements in the megasynthase enzymes as well as substrate predictions based on the phylogeny of KS domains. Module numbering is shown in blue and matches the description given in the main text. Domain abbreviations are as follows: KS, ketosynthase; KR, ketoreductase; DH, dehydratase; ER, enoylreductase; MT, *C*-methyltransferase; Cy, heterocyclization; PS, pyran synthase; GNAT, GCN5-related *N*-acetyltransferase; OM, *O*-methyltransferase; Te, thioesterase; C, condensation; A, adenylation; AL, acyl-CoA ligase. Putative nonelongating KS domains and catalytically inactive DH domains are shaded gray. The key and scale on the lower right refer to all panels. A detailed description of each biosynthetic model is given in the main text.

The structure of compound 1 strongly suggested to us that the molecule is the product of a bacterial *trans*-AT hybrid PKS/nonribosomal peptide synthetase (NRPS) pathway. Structural features that we determined to be particularly useful to identify the BGC for compound 1 include the presence of β-methylations, a thiazole moiety, an *N*,*N*-dimethyl amine, and a rare dilactone-containing macrocycle backbone. Of these, we reasoned that diagnostic sequence features consistent with the incorporation of a thiazole moiety (heterocyclization, cysteine-adenylation, and oxidation domains [40–43]) would be readily identifiable in a short-read sequence assembly, even if the assembly was highly fragmented. Manual analysis of the antiSMASH4 output revealed a putative thiazole-incorporating module that was split over two contigs of 26.6 kb and 39.8 kb in length. The longer of these contigs also included a glycine-activating NRPS module with an associated *N*-methyltransferase that was a feasible origin for the dimethyl amino moiety of pateamine. Moreover, the pattern of methylating, reducing, and dehydrating modules present in this larger contig was consistent with biosynthesis of pateamine A (Fig. 3A). Both contigs were assigned to the *Planctomycetes-Verrucomicrobia-Chlamydiae* (PVC) superphylum and had similar read coverages and GC contents, supporting our presumption that these contigs originated from the same genome. Collectively, these data strongly suggested that we had identified the likely producer of compound 1 within our initial explorative metagenome assembly.

We also expected that we would be able to identify fragments of the mycalamide BGC by virtue of sequence homology with previously solved biosynthetic systems in the pederin family (44–47). A BLAST (tBLASTn) search of the assembly using *pedG* from the pederin BGC as the query sequence returned a single high-identity hit within a 108.9-kb contig. On further investigation, this contig contained a candidate BGC that had a module arrangement nearly identical to that of the onnamide BGC (48) (Fig. 3B) and was therefore likely to encode production of the mycalamides. BLAST searching of nonbiosynthetic genes on the same contig as the presumed mycalamide BGC suggested that the producer was a member of the *Proteobacteria* superphylum, which further indicated that the major components of the secondary metabolome of the sponge could be attributed to distinct prokaryotic producers, neither of which belonged to the metabolically gifted taxon Tectomicrobia.

### Hybrid assembly allows resolution of complete genomes for the dominant microbiome members.

Our initial assembly efforts (MH-Pat-low, Data Set S1) did not resolve the entire pateamine BGC, and employing alternative short-read assembly methods failed to improve the continuity of this genomic region. Furthermore, we were unable to conclusively identify any PKS genes within our assembly that had a module arrangement consistent with production of compound 2. To resolve these issues, we generated long-read data (4.8 Gb, PacBio Sequel) to improve contiguity, as well as additional short-read data (10 Gb, PE150 Illumina) to increase coverage. A hybrid assembly incorporating all short- and long-read data (assembly MH-Pat-all, Data Set S1) was then conducted using the MaSuRCA (49) assembly pipeline, resulting in greatly improved assembly metrics (Fig. 2 and Data Set S1). Contigs from this hybrid assembly were analyzed using antiSMASH4 and assigned to putative genome bins using an ensemble of four binning algorithms (50–53). The improved assembly enabled us to resolve 12 high-quality draft genomes (>85% complete, <15% contamination), 10 of which were nearly complete (>90% complete, <2% contamination), with an average completeness of 96.1% as determined by CheckM. Within the resolved genome bins, the presumed BGC for the mycalamides resided on a 2.7-Mb contig assigned to a 5.8-Mb MAG comprised of 21 contigs. The complete BGC for pateamine was now found on a single contig of 3.09 Mb, located in a MAG containing just two fragments with a total length of 3.10 Mb (Data Set S2).

10.1128/mBio.02997-19.8 DATA SET S2 Quality statistics and taxonomic assignments for each of the high-quality MAGs described in this study. Both 16S rRNA and whole-genome-based taxonomic assignments are presented. Species harboring biosynthetic pathways of interest are highlighted. Download Data Set S2, XLSX file, 0.04 MB. Copyright © 2020 Storey et al. 2020 Storey et al. This content is distributed under the terms of the Creative Commons Attribution 4.0 International license.

Comparison of extracted 16S rRNA sequences from the genomes of both the mycalamide and pateamine producers to publicly available references in the SILVA database (release 132) (54–56) revealed that, in both cases, a new genus name was justified (Data Set S2). The pateamine-producing organism was situated in the *WCHB1-41* family (57) under the phylum *Kiritimatiellaeota* (58, 59). This organism has been assigned the name “*Candidatus* Patea custodiens,” the genus name *Patea* reflecting the historical indigenous population of the area in which the original pateamine-producing sponge was collected and the species name *custodiens* referring to the protective role of the metabolites produced by the organism. The mycalamide-producing organism was situated in the uncultivated UBA10353 marine group order under the *Gammaproteobacteria* phylum. This organism has been assigned the name “*Candidatus* Entomycale ignis,” the species name *ignis* being derived from the Latin word for fire, reflecting the intense skin blistering that arises from exposure to the mycalamides.

### Discovery of the complete pateamine biosynthetic pathway.

The highly contiguous genomes afforded by hybrid assembly enabled us to identify and annotate a complete BGC for the production of pateamine (Fig. 3A). The cluster contains large duplications that were likely the cause of the inability to assemble using short-read data only (Fig. S2). The majority of the biosynthetic genes in the pathway are spread over two separate loci separated by 40 kb of intervening primary metabolic genes. We were also able to identify genes encoding the high-mobility group–coenzyme A (HMG-CoA) synthase and ECH1/2 enzymes necessary for beta-methyl incorporation (24). These were found not in either of the megasynthase loci but rather in a third locus that was 370 kb from the megasynthase regions, which also contains two *trans*-AT domains, as well as machinery for polyketide chain initiation. Based on the observed megasynthase domain arrangement and complement of additional biosynthetic genes, we propose a model in which the cyclic-diester natural product is produced from two separate polyketide chains that are first linked to give a linear ester, followed by macrolactonization to give pateamine (Fig. 3A). The model that we propose is further supported by phylogeny-based predictions of KS domain specificity for the pateamine biosynthetic pathway (Fig. S3).

10.1128/mBio.02997-19.2 FIG S2 Graphical summary of read mapping experiment against the pateamine biosynthetic pathway. Illumina PE150 reads from sample PAT were mapped sequentially from left to right against the pathway. Two large repeat regions (A and B) are observed. Download FIG S2, PDF file, 0.3 MB. Copyright © 2020 Storey et al. 2020 Storey et al. This content is distributed under the terms of the Creative Commons Attribution 4.0 International license.

10.1128/mBio.02997-19.3 FIG S3 Graphical summary of top transATor substrate specificity matches for KS domains in the pateamine biosynthetic pathway. Download FIG S3, PDF file, 0.1 MB. Copyright © 2020 Storey et al. 2020 Storey et al. This content is distributed under the terms of the Creative Commons Attribution 4.0 International license.

### (i) Chain A biosynthesis.

The first module in the pateamine biosynthetic pathway contains a glycine-activating adenylation (A) domain, as well as an *N*-methyltransferase domain. We propose that this module generates *N*,*N*-dimethylglycine and initiates biosynthesis of chain A. Module 2 is a dehydrating module that contains a *C*-methyltransferase, and module 3 is a dehydrating module, consistent with the necessary two α,β-olefinic extensions (the first of which also contains an α-methyl moiety). Module 4 is compatible with incorporation of a β-methyl α,β-olefinic moiety and has three acyl carrier protein (ACP) domains, all of which possess the signature for in-*trans* β-branching enzyme recruitment. Module 5 contains both a dehydratase (DH) and a ketoreductase (KR) domain; however, based on the specificity prediction for the downstream KS domain (Fig. S3) (26, 60) and the presence of a predicted H→D substitution that eliminates the catalytic histidine residue (Fig. S4), we propose that the DH domain is inactive, and this is in fact a β-hydroxy incorporation module. Module 6 contains the expected domain arrangement for incorporation of the observed thiazole moiety, i.e., heterocyclization, adenylation, and oxidation domains. Each of these is present in two copies, and the module also contains three peptidyl carrier protein (PCP) domains. Module 7 contains three β-branching ACP domains, and an enoylreductase, consistent with installation of a fully reduced, β-methylated moiety. Module 8 contains a pyridoxal-5′-phosphate (PLP)-dependent aminotransferase domain and is likely to govern incorporation of the observed β-amino subunit in pateamine to complete chain A.

10.1128/mBio.02997-19.4 FIG S4 Alignment of the putatively inactive DH domain from the pateamine biosynthetic gene cluster against a collection of reference DH domains. The black box indicates the location of an H→D substitution, eliminating the catalytic histidine residue of this domain. Download FIG S4, JPG file, 0.4 MB. Copyright © 2020 Storey et al. 2020 Storey et al. This content is distributed under the terms of the Creative Commons Attribution 4.0 International license.

### (ii) Chain B biosynthesis.

We propose that module 10 is the first module in the biosynthesis of chain B. This module contains a single KS and KR domain but lacks an ACP domain. We suggest that this module acts to reduce ACP-bound β-ketobutyrate and that this is subsequently used to initiate biosynthesis of chain B. Consistent with this suggestion, the producing organism contains genes predicted to encode a type III KS homolog, an ACP domain, and a 4′-phosphopantetheinyl transferase (PPTase) that are clustered with the β-branching and *trans*-AT domains; these likely coordinate to produce ACP-bound β-ketobutyrate from malonyl-ACP and acetyl-CoA. Module 11 contains four consecutive β-ACP domains, consistent with a role as a β-methylating module, and module 12 likely installs a *Z*-configured α,β-olefinic moiety to complete chain B production.

### (iii) Chain transfer, esterification, and macrolactonization.

Module 9 has the domain arrangement ACP_1_-C-ACP_2_ (where “C” represents a condensation domain). This arrangement has previously been observed in malleilactone/burkholderic acid biosynthesis, where it was proposed to tether two separate polyketide chains prior to C-domain-catalyzed condensation to give a linear ester (61–63). We infer a similar role here in pateamine biosynthesis. Specifically, we propose that chain B is transferred from module 11 to ACP_2_ (Fig. 3A, i), and the terminal hydroxyl group of chain B displaces chain A from ACP_1_ in an esterification reaction that is catalyzed by the intervening C-domain (Fig. 3A, ii). In our model, the linear ester is then transferred back to module 13 (Fig. 3A, iii), and the chain is released via thioesterase-catalyzed macrolactonization (Fig. 3A, iv) to give the final macrodiolide.

### Discovery of the complete mycalamide biosynthetic pathway.

Based on structural similarity between mycalamide and onnamide, we expected that the mycalamide BGC would broadly resemble that previously elucidated for onnamide biosynthesis (64). Examination of the antiSMASH4 output for the putative mycalamide-producing bacterium revealed a *trans*-AT polyketide BGC that was nearly identical to that previously described for onnamide (Fig. 3B). The majority of this cluster was found in a single contiguous region on one of the 21 contigs making up the genome of the producing organism. In total, this region encoded all but one of the 12 modules needed to generate the biosynthetic pathway. A second biosynthetic region predicted to encode the initiation module of the megasynthases, the *trans*-AT domains and β-branching enzymes, was located in a separate region of the chromosome.

### Discovery of the peloruside biosynthetic pathway.

In addition to the BGCs for compounds 1 and 3, antiSMASH4 analysis of our hybrid metagenome assembly data revealed a further 24 PKS-containing BGC fragments (14 *cis*-AT and 10 *trans*-AT, Data Set S2). None of these could be conclusively assigned to biosynthesis of compound 2, suggesting that the genome for the producer of this compound was not assembled with sufficient contiguity in our hybrid assembly. In a final attempt to identify this BGC, we conducted an additional short-read-only assembly of the producing sample (MH-PAT) using BBTools (65) for preassembly read merging and the SPAdes (66) assembler, employing both the PE150 and PE250 short-read Illumina data sets. This short-read-only assembly (assembly MH-Pat-SR, Data Set S1) was significantly more fragmented than the hybrid assembly; however, it did yield an additional six draft-quality genome bins that were not present in the hybrid assembly. We examined the antiSMASH4 output from the analysis of MH-Pat-SR and were able to identify a 55.6-kb fragment possessing biosynthetic features that were an excellent match with the structure of compound 2. The presumed peloruside biosynthetic gene cluster contains two large *trans*-AT PKS genes, as well as a standalone acyl-CoA ligase, a standalone ACP, a 2-oxoglutarate-Fe(II)-dependent oxygenase, and an *O*-methyltransferase. There are no *trans*-acting AT domains found in the cluster, and these are likely to be located elsewhere in the genome of the producing organism. The linear order and domain content of modules of the megasynthases, and the additional biosynthetic enzymes present, are in excellent agreement with the known structure of compound 2.

In the model that we propose, the acyl-CoA ligase serves to activate 2-methyl-butanoic acid to the corresponding CoA thioester, which is linked to the freestanding ACP and utilized as a starter unit. Module 1 of the first megasynthase contains a KR and *C*-methyltransferase domain but lacks the expected dehydratase domain (Fig. 3C). We propose that this function is fulfilled by the DH domain present in module 5 and that the intervening modules, which have highly unusual domain architecture, are nonextending. Module 5 then carries out a β-hydroxy extension, and module 6 carries out a β-methoxy extension. Module 7 contains *C*-methyltransferase and KR domains. We propose that the *C*-methyltransferase domain may act twice, and this module carries out an α-dimethyl, β-hydroxy extension. Module 8 then carries out a nonreducing extension, and the resulting carbonyl serves as the electrophile in a spontaneous cyclization reaction to form the pyranose moiety present in the final molecule. The domains of module 9 are consistent with the expected β-methoxy extension. We propose that both modules 10 and 11 incorporate β-hydroxy moieties and that *O*-methylation of the second of these is carried out by the standalone *O*-methyltransferase found in the biosynthetic locus. In our model, each of the noncanonical oxidations is carried out by a 2-oxoglutarate (2OG)-Fe(II)-dependent oxygenase, which could act either on the enzyme-linked intermediates or after release of the macrocycle from the assembly line. Our model contains several unusual extension steps; however, phylogeny-based substrate predictions for the KS domains in the pathway provide additional evidence that our proposal is accurate (Fig. S5). Of particular note is the phylogeny of KS-7, which supports our supposition that the *C*-methyltransferase in the preceding module acts twice to generate the *gem*-dimethyl moiety.

10.1128/mBio.02997-19.5 FIG S5 Graphical summary of top transATor substrate specificity matches for KS domains in the peloruside biosynthetic pathway. Download FIG S5, PDF file, 0.1 MB. Copyright © 2020 Storey et al. 2020 Storey et al. This content is distributed under the terms of the Creative Commons Attribution 4.0 International license.

### Discovery of a new polytheonamide-like gene cluster.

**FIG 4:**
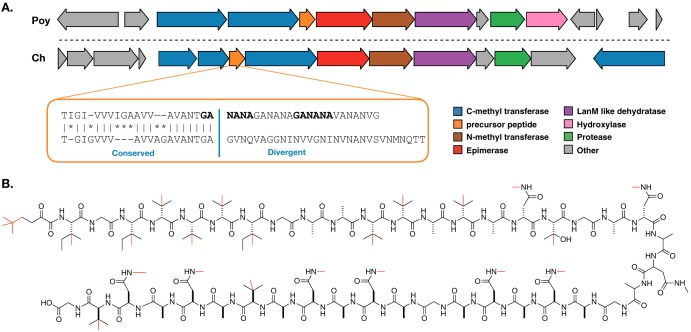
Discovery of a polytheonamide-like RiPP biosynthetic gene cluster. (A) Comparison of the polytheonamide (Poy) biosynthetic BGC and a BGC from the genome of “*Candidatus* Caria hoplita” (Ch) that is predicted to encode a related compound. Genes are colored by predicted function. The inset orange box indicates regions of conservation and divergence between the predicted precursor peptides found in each pathway. (B) Predicted structure for the final product of the novel *M. hentscheli* RiPP cluster. All possible methylations are shown in red; however, it is likely that only a subset of these occur in the final compound.

In addition to the BGCs for compounds 1 to 3, our metagenome assembly contained a number of BGCs that did not appear to match any of the known metabolites previously isolated from *M. hentscheli* (Data Set S3). Of particular interest among these orphan clusters was a 25.0-kb RiPP BGC that appeared to specify a molecule structurally related to polytheonamide (19) (Fig. 4). The architecture and gene content of the BGC are very similar to those of the polytheonamide cluster; however, there are some key differences that indicate it encodes a structurally distinct but functionally related compound. The precursor peptide in this BGC contains a leader sequence which, like that of the polytheonamide precursor, bears homology to the alpha subunit of nitrile hydratases (19). Furthermore, the first 20 amino acids of the core peptide align well with the corresponding residues in the polytheonamide core peptide (Fig. 4A). After residue 20, however, the core peptide sequence diverges markedly from that seen in polytheonamide (Fig. 4A). A particularly striking feature of the core peptide in this BGC is the presence of a hexapeptide motif, GANANA, which is repeated three times in succession.

10.1128/mBio.02997-19.9 DATA SET S3 Summary of unique biosynthetic gene clusters recovered from all assemblies. Where applicable, MAG of origin is indicated; UB indicates clusters that were found on unbinned contigs. The cluster numbers match those in the antiSMASH output that can be downloaded from https://github.com/MaxMeta/MH_project_data. Download Data Set S3, XLSX file, 0.04 MB. Copyright © 2020 Storey et al. 2020 Storey et al. This content is distributed under the terms of the Creative Commons Attribution 4.0 International license.

The spacing of achiral residues (glycines) in the core, coupled with the presence of a PoyD-like epimerase, suggests that, like polytheonamide, our RiPP product likely possesses d-configured or achiral residues at every second position. The sequence of the core peptide, coupled with the content of tailoring enzymes in the cluster, allowed us to make a prediction (with some uncertainty) for the final structure of the encoded metabolite, which is presented in Fig. 4B. The BGC is found within a 7.0-Mb MAG with an *N*_50_ of 4.7 Mb and contains an additional 9 putative BGCs (Data Set S4). The extracted 16S sequence from this MAG positions the producer strain for this RiPP as a new genus, within the family *Nitrosococcaceae*, which falls under the *Gammaproteobacteria* phylum (Data Set S2). The name “*Candidatus* Caria hoplita” has been assigned to this species.

10.1128/mBio.02997-19.10 DATA SET S4 Taxonomic assignments for unique 16S rRNA genes recovered across all metagenome assemblies conducted as part of this study. Download Data Set S4, XLSX file, 0.04 MB. Copyright © 2020 Storey et al. 2020 Storey et al. This content is distributed under the terms of the Creative Commons Attribution 4.0 International license.

### Comparative community analysis for five *M. hentscheli* specimens.

**FIG 5:**
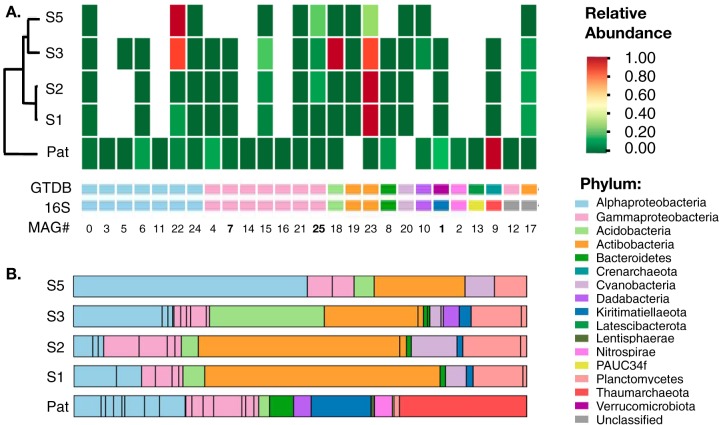
Microbiome comparison for the five *M. hentscheli* specimens. (A) Heat map indicating relative abundances for each of the 26 high-quality MAGs elucidated in this study with rows clustered by similarity. The lower strips indicate the phylum of each MAG as deduced by either 16S rRNA sequence analysis (16S) or whole-genome-based taxonomy (GTDB). Numbering at the bottom of the panel matches the MAG numbering in Data Sets S2 to S4. The bold numbers indicate “*Candidatus* Patea custodiens” (MAG 1), “*Candidatu*s Entomycale ignis” (MAG 7), and “*Candidatus* Caria hoplita” (MAG 25). (B) Phylum-level microbiome composition for each of the five specimens examined in this study as deduced by extracting 16S sequences directly from metagenome assemblies. Abundance values were derived from coverage of the corresponding contig in the assembly. Black bars within the same colored block denote multiple species within the same phylum.

Having obtained a high-quality reference hybrid assembly from the microbial metagenome of a single *M. hentscheli* specimen (MH-PAT), we next sought to examine the temporal and spatial variability of the *M. hentscheli* microbiome using metagenome sequence data from an additional four specimens. These specimens were collected in 2003 from a different location and exhibited different chemotypes than the sample used for the initial reference assembly (Fig. 1). Approximately 10 Gb PE150 Illumina sequence data were collected for each additional sample. The resulting read sets were preprocessed using BBTools and then assembled individually using SPAdes. Metagenome binning of each assembly was carried out using an ensemble of four algorithms (50–53), and the collection of genome bins computed across all five samples was dereplicated (67) to generate a final set of 26 unique high-quality MAGs (>85% completeness and <15% contamination). We were able to identify partial or complete 16S genes for 24 of these MAGs, and these were used for taxonomic assignment by comparison to the SILVA database (55, 56). The final MAGs were also analyzed using the GTDB-Tk pipeline (v 0.3.2) (68, 69) to generate whole-genome-based taxonomic classifications (70, 71) for NCBI genome submission (Fig. 5 and Data Set S3). The classifications assigned to the MAGs by the two methods were highly congruent (Fig. 5 and Data Set S3).

In order to obtain relative abundance data for each of these putative MAGs across the pan-metagenome assemblies, we mapped the reads from each of our five specimens against all of the assemblies. This analysis revealed that the identity of the species present in each of our five samples of *M. hentscheli* was remarkably stable (Fig. 5A). Of the 26 high-quality MAGS assembled across the pan-metagenome, 17 were seen in at least four of the five specimens, an observation that suggests that *M. hentscheli* stably maintains a defined microbiome that contributes cooperatively to the secondary metabolic output of the holobiont. Manual examination of the antiSMASH4 outputs for the putative “*Candidatus* Caria hoplita,” “*Candidatus* Patea custodiens,” and “*Candidatus* Entomycale ignis” MAGs, across each sample in which they were found, revealed that in each case the BGCs for mycalamide, pateamine, and the putative polytheonamide-like RiPP were present in the expected bin. This further supports our assignment of these strains as producers of their respective metabolites.

The presence of microbial species to which we attribute previously characterized secondary metabolites does not strictly correlate with detection of the respective secondary metabolite. In particular, “*Candidatus* Patea custodiens” was present at relatively high levels in four of our specimens (Fig. 5A); however, only one of these samples had sufficient levels of pateamine for detection using our ^1^H NMR spectroscopic analysis. The contiguity of the “*Candidatus* Patea custodiens” genome from each of these samples was variable; however, mapping reads from each sample against the complete pateamine biosynthetic pathway indicated that in each case, the biosynthetic loci were complete (>99% coverage). This suggests that although *M. hentscheli* maintains a stable cohort of microbes, in some cases this chemical potential is latent and awaiting an appropriate environmental cue.

Another striking feature was the relative simplicity of individual microbiomes that were consistently dominated by just a few (<4) high-abundance species (Fig. 5 and Fig. S6). Outside of the few dominant species in each sponge, the rest of the microbiome showed a precipitous drop in relative coverage with an approximately 200-fold range of abundance overall. This is in contrast to other sponge microbiomes that display a more complex and flatter distribution of species (72–74).

10.1128/mBio.02997-19.6 FIG S6 Relative proportions and phylum-level taxonomy for the 15 most abundant 16S-containing contigs recovered from each metagenome assembly. Download FIG S6, PDF file, 0.04 MB . Copyright © 2020 Storey et al. 2020 Storey et al. This content is distributed under the terms of the Creative Commons Attribution 4.0 International license.

By extracting 16S rRNA gene sequences directly from our unbinned assemblies, we were able to tentatively identify an additional 24 unique microbial species (clustered at 97% identity) that did not map to any of our recovered MAGs (Fig. 5 and Data Set S4). The contigs from which these sequences arose had low coverage, indicating that they might arise from transient or incidental microbiome members. While this analysis allowed us to identify additional microbiome members, it should be noted that recovery of 16S sequences from metagenome assemblies can be problematic, and it is likely that additional species were present in our samples. However, none of the partial or complete 16S rRNA sequences that we recovered from either assemblies or unbinned contigs suggested the presence of members of the phylum Tectomicrobium, and whole-genome taxonomic analysis of our MAGs did not identify any potential members of this phylum in our samples (Fig. 5A).

### Concluding remarks.

We employed an untargeted, informatics-driven discovery strategy to examine the microbial consortia associated with five different specimens of *M. hentscheli*. By using hybrid assembly of Illumina and PacBio data, coupled to an ensemble of metagenome binning algorithms, we were able to elucidate highly contiguous, nearly complete MAGs for the dominant microbiome members. Comparison of microbiome composition among five individuals collected at two separate sites revealed that the identity of prokaryote species present was highly conserved; however, their relative abundances varied widely between sponge isolates.

Our approach enabled us to identify complete BGCs for each of the cytotoxic polyketides previously isolated from *M. hentscheli*, without any prior knowledge of the localization, morphology, or phylogeny of producing organisms. This work follows from observations that members of the genus *Entotheonella* are the dominant producers of cytotoxic polyketides in a number of marine sponges. In the case of the most extensively studied species, Theonella swinhoei, the majority of the chemistry associated with a particular chemotype can be traced to a single metabolically gifted symbiont, and variations in chemotype are attributable to variations in the identity of the *Entotheonella* symbiont species present. The genus *Mycale* shares many characteristics with the Theonellidae sponges, namely, a rich and varied secondary metabolome and the existence of multiple chemotypes within a single species. However, in *M. hentscheli* no evidence was found to suggest the presence of microbes belonging to the genus *Entotheonella*. Instead, our data suggest a possible cooperative mode of symbiosis in which the secondary metabolome of *M. hentscheli* is sculpted by multiple uncultivated bacterial producers, each harboring a relatively modest collection of BGCs. Functional assignments in this work are based on comparison to other known biosynthetic systems; however, it will be necessary in this, and other cases, for biochemical characterization to be undertaken in order to confirm putative functional assignments.

Three of the producing species that we identify are the founding members of newly defined bacterial genera *Entomycale*, *Patea*, and *Caria*. The presence of both onnamide- and polytheonamide-like gene clusters directly parallels the situation in *T. swinhoei*; however, in *M. hentscheli* these two clusters are hosted by symbionts that are phylogenetically distant from the *T. swinhoei* producer (Entotheonella factor) (23). The observation of similar BGCs, playing similar roles in such distantly related microbial species, emphasizes the extreme horizontal migration of this cluster and contributes to a growing body of work that suggests that acquisition of BGCs encoding compounds with potential defensive properties might drive the formation of stable long-term associations between hosts and their microbial symbionts.

## MATERIALS AND METHODS

### Sample collection.

Marine sponges of the species *M. hentscheli* (*n* = 5) were collected by scuba at two locations in the Marlborough Sounds, South Island, New Zealand. Sample “MH-PAT” was collected from a depth range of 5 to 15 m at Capsize Point in November 2014. The four additional specimens of *M. hentscheli* included in this study (designated s1, s2, s3, and s5) were collected during a separate expedition from a depth range of 5 to 15 m at Pelorus Sound in May 2003.

### Chemotyping.

Frozen sponge samples were homogenized by grinding under liquid nitrogen. The powdered sponge samples (∼1.5-g dry weight) were then extracted with 80% MeOH-H_2_O (20 ml) and then MeOH (20 ml) for 10 min each. The first extract, followed by the second extract, was passed through a polystyrene divinylbenzene (PSDVB) column (2 ml). The combined eluents were diluted and reapplied to the column a total of three times, using H_2_O for dilution (2 ml twice, then 80 ml). The column was eluted with H_2_O (10 ml) and then 55% Me_2_CO-NH_4_ acetate (OAc) (0.2 M, adjusted to pH 4 with AcOH). The latter fraction was neutralized with NH_4_OAc (0.2 M, 20 ml) and then loaded onto another PSDVB column (0.5 ml) to desalt and remove water. The fraction was passed through the column twice, followed by elution with H_2_O (10 ml) and then Me_2_CO (6 ml). The resulting Me_2_CO fractions were dried and analyzed by ^1^H NMR and LC-MS. ^1^H NMR spectra were acquired using a 600-MHz Varian Direct Drive spectrometer. Spectra were recorded in CDCl_3_ and referenced to the residual solvent peak (δ_H_ 7.26). LC-MS data were acquired with an Agilent 6530 accurate-mass quadrupole time of flight (Q-TOF) LC-MS mass spectrometer equipped with a 1260 Infinity high-pressure liquid chromatography (HPLC) system using positive-mode electrospray ionization. The instrument parameters were set as follows: gas temperature, 275°C; drying gas, 9 liters/min; nebulizer, 30 lb/in^2^; sheath gas temperature, 300°C; sheath gas flow, 10 liters/min; capillary voltage, 4,000 V; nozzle voltage, 500 V. Masses were recorded between 100 and 2,000 *m/z* at a rate of 3 spectra per second. Chromatographic separation was achieved with a reversed-phase C_18_ column (Kinetex; 50 mm by 2.1 mm by 2.6 μm), set to 35°C. Sample elution was achieved using eluent A (H_2_O-0.1% HCO_2_H) and eluent B (acetonitrile [ACN]-0.1% HCO_2_H) with a gradient from 5% B to 100% B over 11 min at a flow rate of 0.4 ml/min. Samples were adjusted to a concentration of 0.1 mg/ml in methanol (MeOH), and an injection volume of 10 μl was used.

### Metagenomic DNA extraction.

Metagenomic DNA for both Illumina and PacBio sequencing was extracted from frozen sponge samples homogenized by grinding under liquid nitrogen. Ground sponge tissue from a longitudinal section capturing both pinacoderm and mesohyl was suspended in NTE buffer (500 mM NaCl, 100 mM Tris-HCl [pH 8.0], 10 mM EDTA) and vortexed to dissociate prokaryotic cells. The prokaryotic cell fraction was then enriched by centrifugation of the ground suspended sponge tissue at 50 × *g* for 45 s to pellet and remove sponge tissue. The supernatant was then transferred to a fresh tube and centrifuged at 3,100 × *g* for 10 min to pellet the remaining prokaryote-enriched fraction. This cell pellet was suspended in an equal volume of NTE buffer before the addition of 10 volumes of fresh sponge lysis buffer (75) (8 M urea, 2% Sarkosyl, 1 M NaCl, 50 mM EDTA, and 50 mM Tris-HCl, pH 7.5). Lysis was achieved by incubation at 50°C for 60 min with gentle agitation, and the resulting lysate was extracted twice with an equal volume of 1:1 phenol-chloroform. DNA was recovered from the aqueous phase by isopropanol precipitation, and high-molecular-weight (HMW) DNA (>23 kb) was purified and size selected by agarose gel electrophoresis. DNA samples were recovered from gel slices by electroelution, and purified DNA was stored in TE buffer at 4°C. At no point was the DNA exposed to DNA stains or UV irradiation. For sample MH-PAT, where multiple read sets were generated, each of these was generated from a single metagenomic DNA extraction.

### Sequence data generation. (i) PE250 Illumina data.

A PCR-free Illumina TruSeq library with an average insert fragment size of 800 bp was prepared from the MH-PAT-derived metagenomic DNA and sequenced on the HiSeq 2500 platform. Library preparation and data acquisition were carried out by Novogen Inc., China.

### (ii) PacBio data.

Long-read sequence data were generated from >15 μg of HMW MH-PAT metagenomic DNA on the PacBio Sequel system using one single-molecule real-time sequencing (SMRT) cell. Library preparation and data acquisition were carried out by Macrogen Inc., Seoul, South Korea.

### (iii) PE150 Illumina data.

For each *M. hentscheli* sample in this study (MH-PAT, s1, s2, s3, and s5), an Illumina TruSeq library with an average insert size of 500 bp was prepared from extracted metagenomic DNA and sequenced on the HiSeq 4000 platform. Library preparation and data acquisition were carried out by Genewiz, Suzhou, China.

### Metagenome assembly.

For the initial investigative short-read assembly (MH_Pat_sr) of the metagenome of the PAT sponge sample, PE250 Illumina reads were processed prior to assembly using Skewer (v0.2.2) (76) for quality and adapter trimming with a Phred cutoff of Q30. An assembly was then calculated from the trimmed reads using a customized version of IDBA-UD (v1.1.1) (37) modified to allow a –maxk length of 250 to be used.

The coassembly of the two short data sets (MH_Pat_All_sr_spades_merge) of the MH-PAT sample (PE250 and PE150) was conducted using SPAdes (v3.13.0) (66). The reads were preprocessed to remove adapter sequences and merged using BBTools (v38.08) (65) prior to assembly as follows: reads were adapter trimmed and decontaminated by kmer matching to Illumina adapters, PhiX sequence, and masked versions of human (HG19) and common laboratory microbe sequences. Reads were quality trimmed to Q10, and any reads containing ambiguous base calls (N > 0) were removed.

The hybrid short- and long-read assembly for the MH-PAT sponge (MH-Pat_all) was computed using MaSuRCA (v3.2.8) (77). This assembly was produced from the unmodified reads of all three data sets collected from this sample (PacBio, PE250, and PE150).

Metagenome assemblies for samples s1, s2, s3, and s5 were computed from the respective PE150 reads as follows. The BBTools package (v38.08) was used as already described. The reads were then error corrected, and any overlapping read pairs were merged into a single read. The preprocessed data sets were assembled using SPAdes (v3.12.0). Only contigs of >2,000 bp from assemblies were used for further analysis.

Further details of read processing and parameters used in the assemblies are contained in scripts available at github.com/Mattstorey/MH_sponge/MH_assembly. Summary statistics for each assembly are given in Data Set S1 in the supplemental material.

### Contig binning and resolution of MAGs.

Four automated binning algorithms, MaxBin 2 (51), Concoct (53), MetaBAT 2 (52), and Autometa (50), were run individually to extract bins from each metagenomic assembly. MaxBin 2, Concoct, and metaBAT 2 were implemented in the binning module of metaWRAP and were provided with quality and contaminated filtered PE150 read sets to inform differential coverage profiles. Autometa was run from the Docker image provided at https://hub.docker.com/r/jasonkwan/autometa. Bacterial contigs were isolated based on taxonomic kingdom, and then unclassified contigs were recruited with the “supervised machine learning” option. The required coverage file for each sample was derived from the BBmap covstat output for the alignment of the PE150 read set against its respective metagenome assembly. The outputs of all binning methods against all samples were combined, resulting in 1,262 identified bins. Dereplication was then carried out using DRep (67), resulting in a set of 26 prokaryote bins with a CheckM (78) cutoff of >85% completeness and <15% contamination.

### Detection and annotation of biosynthetic gene clusters.

Secondary-metabolite BGCs in the metagenomic assemblies and bins were detected and annotated using a standalone Docker implementation of antiSMASH4 (38) (wrapper script provided at https://bitbucket.org/antismash/docker/raw/HEAD/standalone/run_antismash).

KS domain specificities were predicted using the transATor (26) web tool (https://transator.ethz.ch/). Assignment of gene function for the mycalamide, peloruside, pateamine, and polytheonamide-like RiPP BGCs was further facilitated by BLASTP and conserved-domain searches of amino acid sequences from open reading frames in each cluster. Dereplication of biosynthetic gene clusters to generate a final set of unique gene clusters was achieved using the average nucleotide identity clustering facility of the DRep package. This process is outlined in the Jupyter notebook available at github.com/Mattstorey/MH_sponge/MH_BGCs.

### Assignment of phylogeny to metagenome-assembled genomes.

For the data presented in Fig. 2, taxonomy was assigned to individual contigs from the MH_Pat_sr and MH_Pat_all metagenome assemblies as described in the work of Albertsen et al. (39). Briefly, open reading frames were called for all contigs in an assembly with Prodigal (79), and the single-copy marker proteins were identified using HMMER 3.0 (80) (v 3.1b2) with an established set of 113 hidden Markov models (HMMs) for conserved essential genes. The identified marker protein sequences from each contig were queried against the NCBI refseq database (refseq_protein v 83). A phylum level taxonomy was extracted from the BLAST results of each marker using the least common ancestor (LCA) output of MEGAN6 and assigned to the originating contig. If multiple marker proteins were detected in a single contig, final taxonomic assignments were based on majority vote consensus. Scripts and marker genes used for this analysis can be accessed at github.com/MadsAlbertsen/multi-metagenome.

For 16S rRNA-based taxonomic classification, full-length and partial 16S genes were extracted from MAGs as well as unbinned contigs using Barrnap (v 0.9) (github.com/tseemann/barrnap) and dereplicated using the Dedupe module of BBTools (v38.08) with minidentity = 97. These were taxonomically classified using the SILVA (56) rRNA database project (www.arb-silva.de/aligner/). The 16s rRNA gene sequences were searched against the SILVA database with default setting using the LCA method and a minimum identity of 0.85 against all available taxonomies. For whole-genome-based taxonomic classification, the classify workflow of GTDB-Tk (v 0.3.2) (68, 69) was used.

### Detection of Tectomicrobia in metagenome data.

No extracted 16S rRNA gene sequences were classified as Tectomicrobia, the parent phylum of the genus “*Candidatus* Entotheonella.” To confirm these findings, short reads were mapped to all available (six) taxonomically labeled Tectomicrobia genomes from NCBI using BBsplit of BBTools (v38.08) (65) with minidentity = 97. This resulted in no significant mapping to the reference genomes, with only a few hundred reads of 10 million sparsely mapping across all six genomes. All reads that did map were aligned to two short regions on a single contig, designated “an unlocalized plasmid scaffold,” from the metagenome-derived assembly of “*Candidatus* Entotheonella” sp. TSY1. A BLASTn query of the 2-nt sequence where the mapped reads aligned against the nr/nt database suggested that these regions are 28S ribosomal sequence from sponges. These results suggest no significant detectable presence of species in the Tectomicrobia phylum.

### Abundance profiling of MAGs.

For the data presented in Fig. 5, short-read data set PE150 data for each sample (MH_Pat and s1, s2, s3, and s5) were quality and adapter filtered with BBDuk, and each filtered read set was individually mapped against the assemblies (MH_Pat_all and s1, s2, s3, and s5) using BBMap. Coverage statistics for the contigs of each assembly were generated across all samples by enabling the covstat flag. Data were processed and visualized using Python scripts which have been packaged as a Jupyter notebook; these can be found at github.com/Mattstorey/MH_sponge/MH_phylo.

### Data availability.

All metagenome assemblies, read sets, and MAGs described in this publication have been deposited in NCBI. Accession numbers for metagenome assemblies are SAMN12718690 to SAMN12718694. Accession numbers for individual high-quality MAGs are SAMN12903678 to SAMN12903703. Read sets have been deposited in SRA as SRR10090292, SRR10090327 to SRR10090331, and SRR10220753. Further details are given in Data Sets S1 and S2.
