# Supplementary figures and images for: Metagenomic Exploration of the Marine Sponge Mycale hentscheli Uncovers Multiple Polyketide-Producing Bacterial Symbionts
Source: mBio. 2020 Mar 24;11(2):e02997-19. doi: 10.1128/mBio.02997-19 (PMC7157528; doi:10.1128/mBio.02997-19)

A.

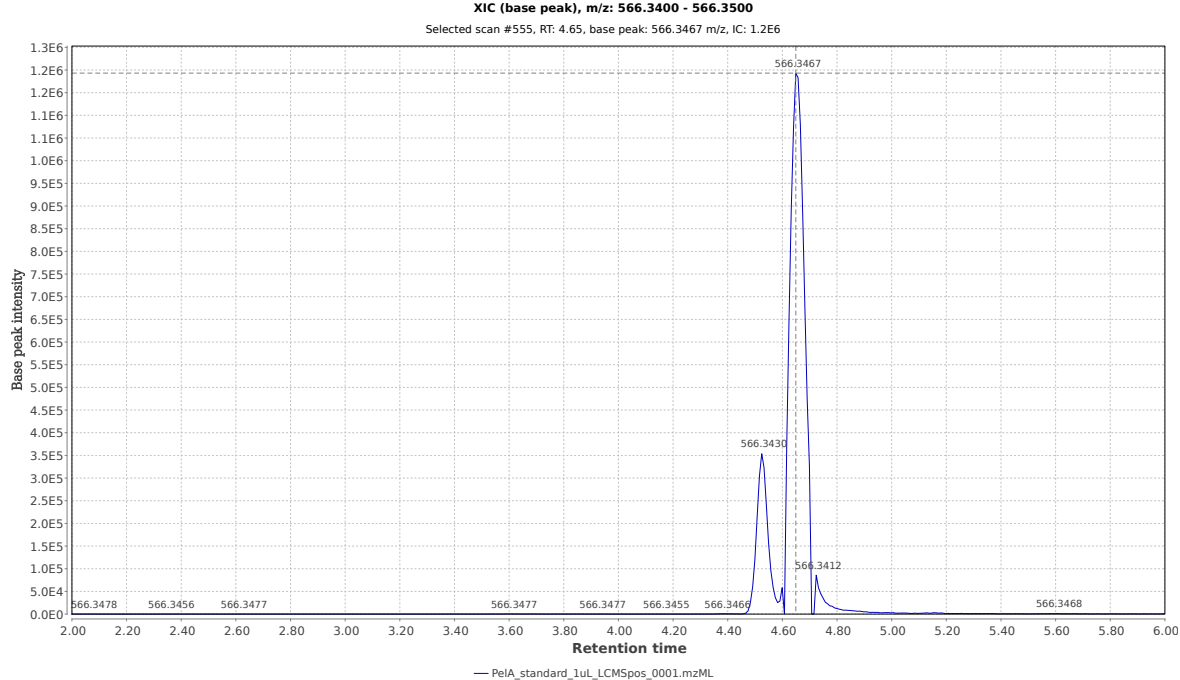

B.

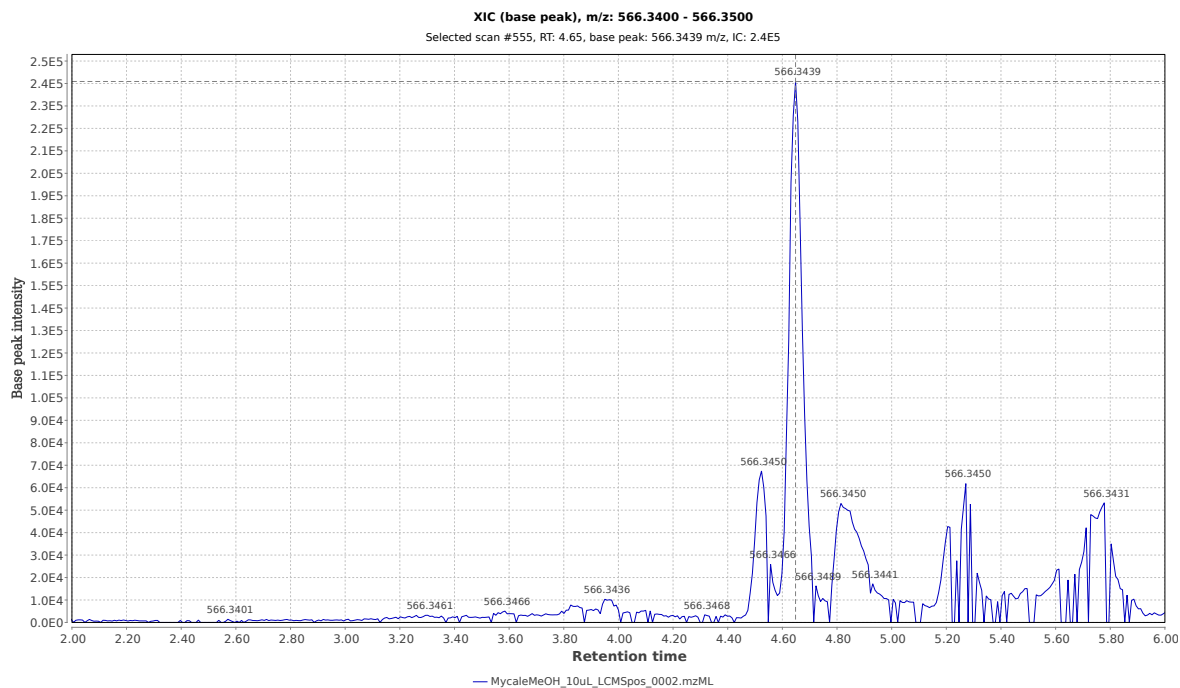

Supplement: FIG S1 [file mBio.02997-19-sf001.pdf]

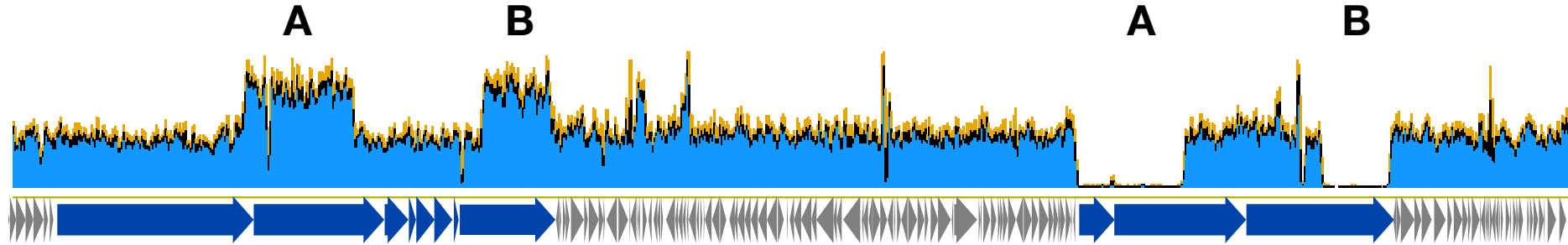

Supplement: FIG S2 [file mBio.02997-19-sf002.pdf]

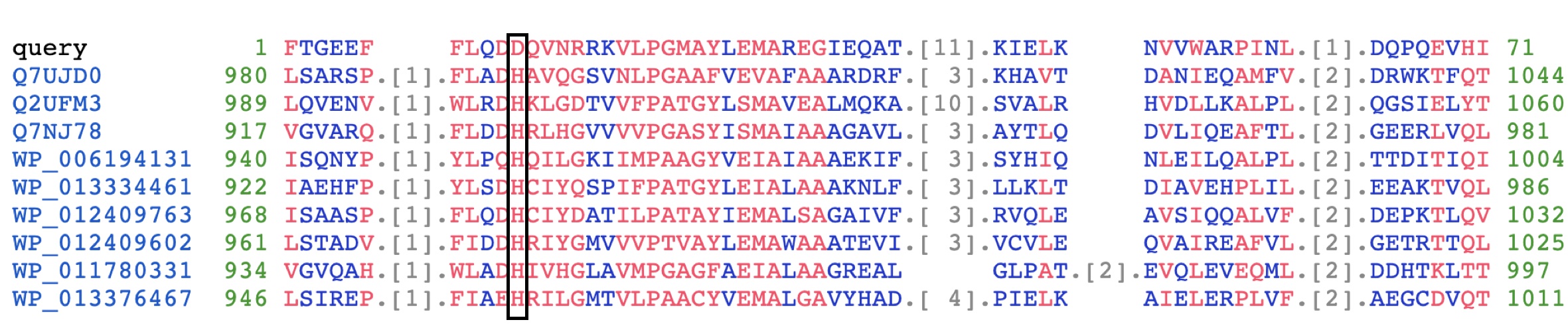

Supplement: FIG S4 [file mBio.02997-19-sf004.jpg]

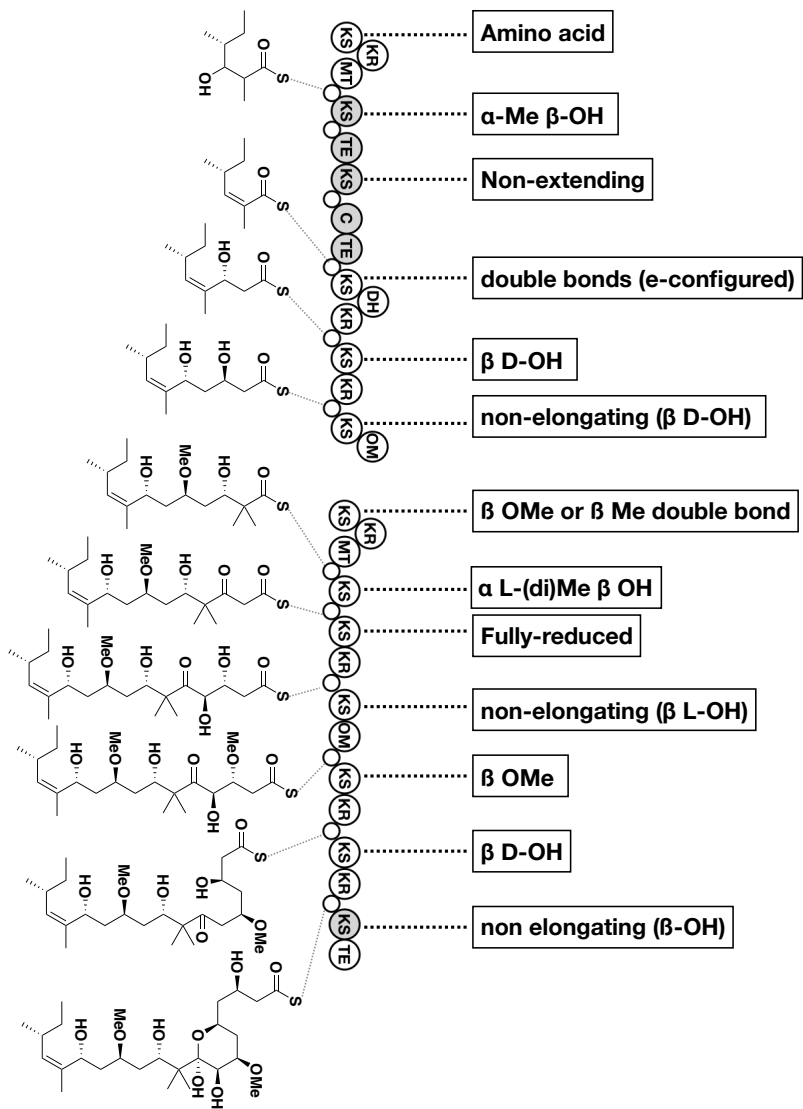

Supplement: FIG S5 [file mBio.02997-19-sf005.pdf]

PAT

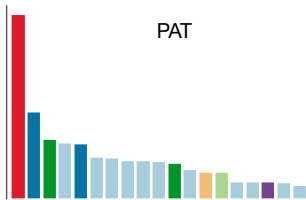

S3

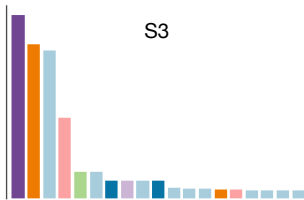

S1

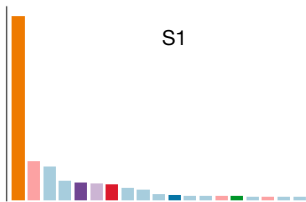

S5

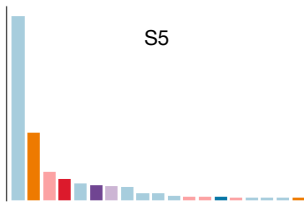

S2

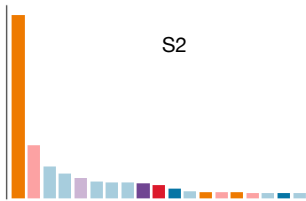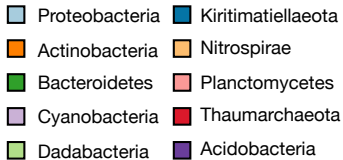

Supplement: FIG S6 [file mBio.02997-19-sf006.pdf]
