# Supplementary material for: Metagenomic Exploration of the Marine Sponge Mycale hentscheli Uncovers Multiple Polyketide-Producing Bacterial Symbionts
Source: mBio. 2020 Mar 24;11(2):e02997-19. doi: 10.1128/mBio.02997-19 (PMC7157528; doi:10.1128/mBio.02997-19)
Supplement: FIG S3 [file mBio.02997-19-sf003.pdf]

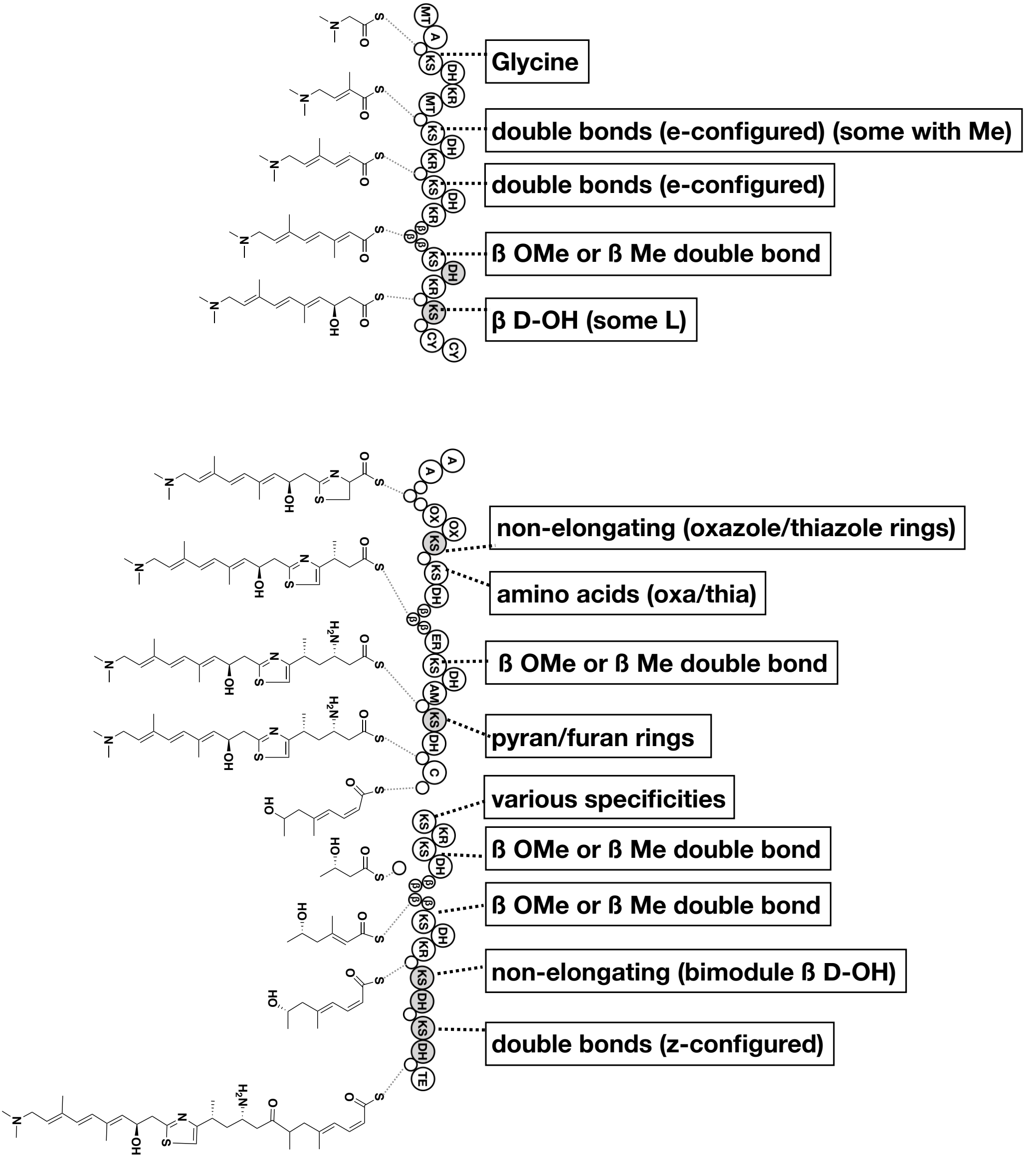

Glycine
double bonds (e-configured) (some with Me)
double bonds (e-configured)
ß OMe or ß Me double bond
ß D-OH (some L)
non-elongating (oxazole/thiazole rings)
amino acids (oxa/thia)
ß OMe or ß Me double bond
pyran/furan rings
various specificities
ß OMe or ß Me double bond
ß OMe or ß Me double bond
non-elongating (bimodule ß D-OH)
double bonds (z-configured)
